# Enhancing bariatric surgery safety for patients refusing blood transfusions: a specialized protocol with comprehensive technical measures

**DOI:** 10.1007/s13304-024-01912-5

**Published:** 2024-06-12

**Authors:** Muhammed Said Dalkılıç, Mehmet Gençtürk, Abdullah Şişik, Hasan Erdem

**Affiliations:** 1 Department of General Surgery, Marmara University School of Medicine, Istanbul, Turkey; 2 Dr HE Obesity Clinic, Istanbul, Turkey

**Keywords:** Jehovah’s witnesses, Bariatric surgery, Blood transfusion, Sleeve gastrectomy, Roux-en-Y gastric bypass, Bloodless surgery

## Abstract

Bariatric surgery has become a leading treatment for obesity, with techniques such as Laparoscopic Sleeve Gastrectomy (LSG) and Laparoscopic Roux-en-Y Gastric Bypass (LRYGB) demonstrating notable success in sustained weight loss and improved quality of life. Technological advancements and improved techniques have enhanced the safety of these procedures. The surgical procedures of Jehovah’s Witnesses, who refuse blood transfusions as part of their beliefs, pose unique challenges and have rarely been addressed in the context of bariatric surgery. This report aimed to investigate the safety of bariatric surgery in patients who refuse blood transfusion, with an established protocol to minimize the risk of bleeding. We examined the prospectively collected data of Jehovah’s Witness patients who underwent bariatric surgery from 2019 to 2023. The surgeries were conducted following a protocol that included specific measures to prevent bleeding. Data were reviewed for demographics, anthropometrics, comorbidities, preoperative medications, operative time, blood loss, length of hospital stay, hemoglobin level, drainage volume, tranexamic acid use, and postoperative 30-day complications. Eleven Jehovah’s Witness patients underwent bariatric surgery, including 10 LSG and 1 LRYGB. A patient with iron deficiency anemia underwent intravenous iron treatment before the surgery. There were no intraoperative complications or major postoperative complications. All patients maintained stable hemodynamics postoperatively. Only one patient encountered nausea–vomiting, classified as a minor complication. One patient experienced a small amount of hemorrhagic drainage, which transitioned to serous after tranexamic acid infusion. Bariatric surgery can be performed safely with established protocols in patients who refuse blood transfusions.

## Introduction

Bariatric surgery has emerged as the forefront treatment for obesity, experiencing rapid global expansion. The surgical techniques such as Laparoscopic Sleeve Gastrectomy (LSG) and Laparoscopic Roux-en-Y Gastric Bypass (LRYGB) have demonstrated remarkable success in achieving sustained weight loss, improved survival and quality of life, and the effective management of obesity-related comorbidities [1]. Notably, LSG has become the most widely performed bariatric procedure globally, owing to its technical simplicity, short learning curve, and efficacy [2].

Technological advancements and refined surgical techniques have significantly improved the safety profile of bariatric surgery. Recent studies show a significant reduction in hemorrhagic complication rates (0.4–1.3%), the most common early complication after bariatric surgery [3].

Certain religious groups, such as Jehovah’s Witnesses, hold beliefs that prohibit blood transfusion. With approximately 8 million members globally and 1 million in the United States, Jehovah’s Witnesses adhere to the belief that carrying the blood of another person is forbidden by God, grounded in specific statements in the Bible [4]. The three biblical passages that forbid the consumption of blood (Genesis 9:4, Leviticus 17:10–14, Deuteronomy 12:23–25, Acts 15:29, and Acts 21:25) are also interpreted to prohibit the use of blood products. It is widely acknowledged that transfusion of whole blood, packed red blood cells, plasma, and platelets is prohibited. However, the use of other treatments such as cryoprecipitate, hemostatic complex concentrates, and fibrin sealant products is left to individual discretion. According to JW belief, failure to comply with this principle may result in forfeiting eternal salvation [4]. This refusal of blood product transfusion has presented unique challenges, especially for surgeons and anesthesiologists, necessitating specific considerations.

While acknowledging the patients’ right to reject recommended treatments, including blood transfusions, additional measures have been implemented to mitigate bleeding risks. Special protocols have been established in high-volume centers to address the distinctive needs of Jehovah’s Witnesses [5, 6]. This situation has been widely discussed in cardiac surgery where blood product transfusions are common [4], but has rarely been addressed in bariatric surgery. The primary aim of this report is to investigate the safety of a perioperative protocol for bariatric surgery in this unique cohort of patients who refuse blood transfusion.

## Methods

Between 2019 and 2023, a specialized protocol was implemented to minimize bleeding risk in patients scheduled for bariatric surgery who refused blood product transfusions due to religious reasons. This study presents a retrospective analysis of prospectively collected data. We received approval from the institutional review board. All patients provided informed consent for the surgical procedure following a comprehensive and clear elucidation of the associated risks, particularly in terms of bleeding and potential life-threatening consequences resulting from the refusal of a blood transfusion. Also, all patients provided informed consent for the collection and utilization of their data for research purposes.

Initially, the surgical team provided detailed information to all patients regarding the advantages, disadvantages, risks, and benefits of the procedures. Subsequently, the multidisciplinary council recommended specific procedures based on targeted weight loss, the patient’s lifestyle, and medical history. However, the ultimate decision rested with the patient.

Data from a prospective database of all patients undergoing bariatric surgery were retrospectively reviewed for demographics, anthropometrics, comorbidities, preoperative medications, operative time, estimated blood loss, length of hospital stay, hemoglobin level (preoperative and postoperative), drainage volume, tranexamic acid use, and postoperative first 30-day complications. Preoperative risks were categorized based on the American Society of Anesthesiologists’ physical status classification system. The complications were categorized according to theClavien–Dindo Classification. Descriptive statistics, including mean and standard deviation, were employed to present the data.

**Fig. 1:**
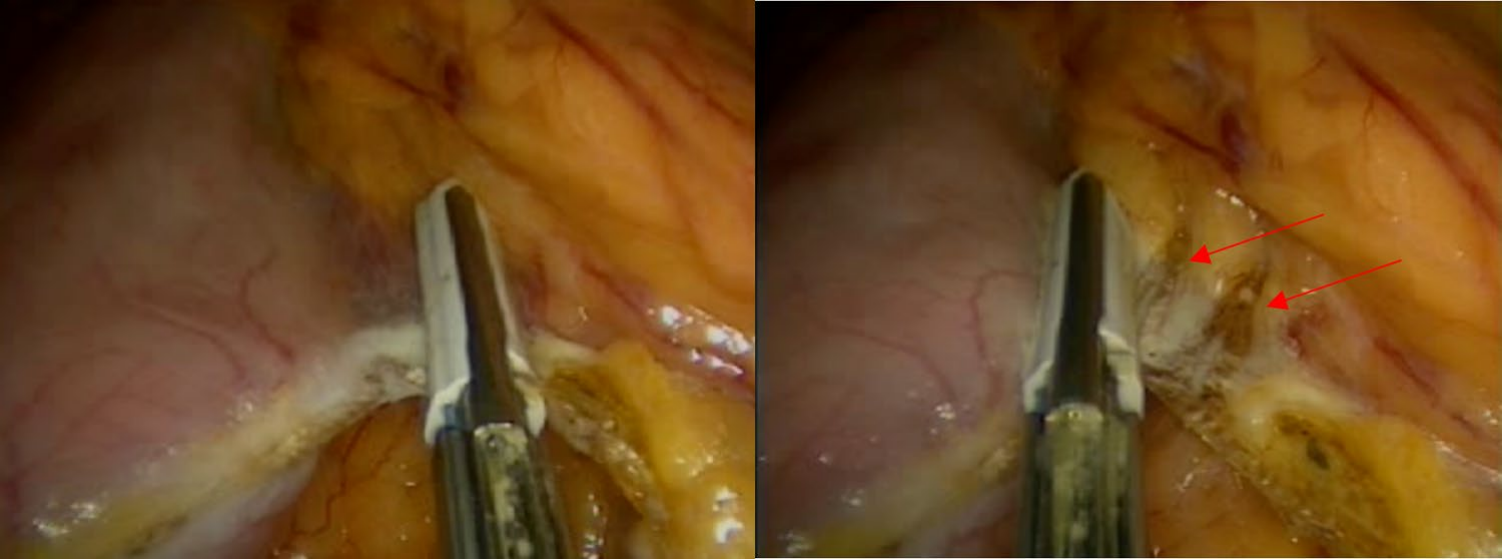
Double line sealing technique: sealing vessels in two adjacent rows during omental dissection to minimize bleeding risk in LSG. The arrows indicate the sealed lines on the omentum

**Fig. 2:**
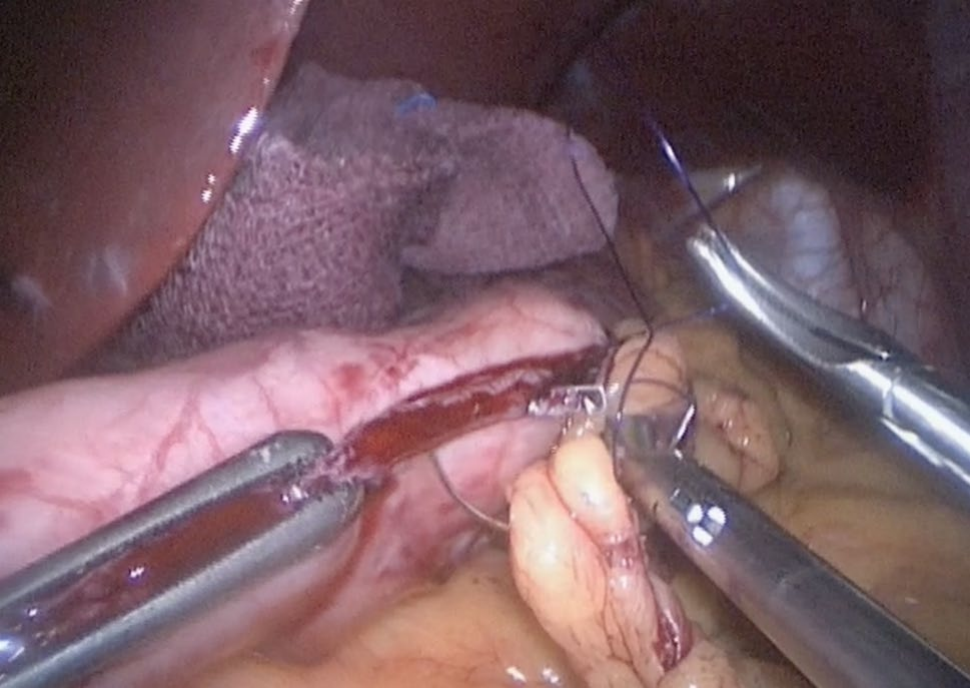
Staple line oversewing With omentopexy in LSG

**Fig. 3:**
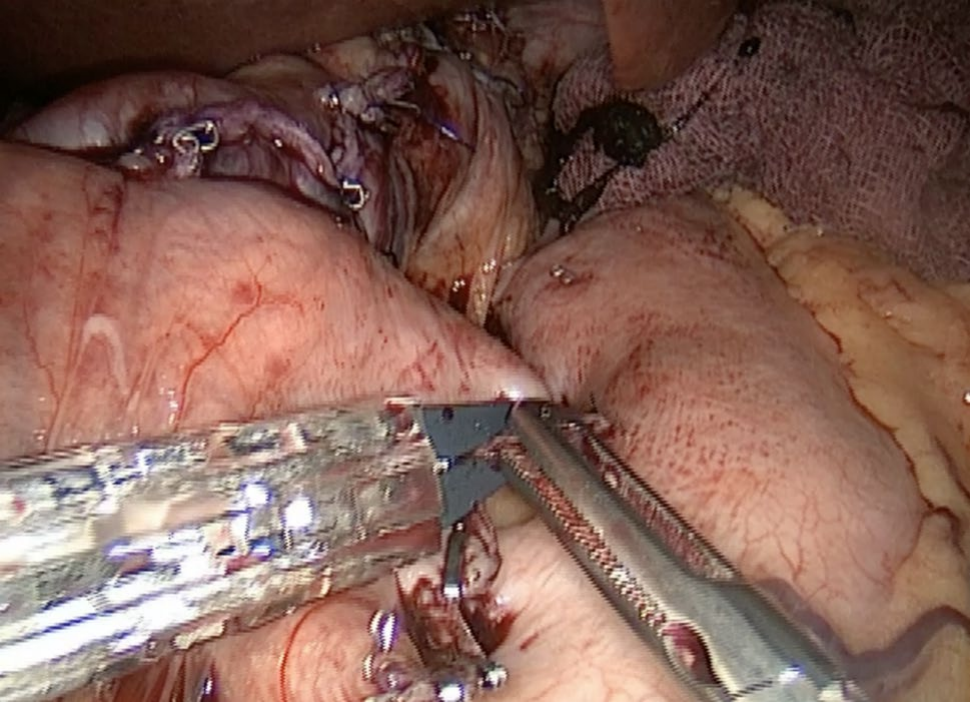
Application of hemostatic clips to potential bleeding sites along the staple line during LRYGB

**Fig. 4:**
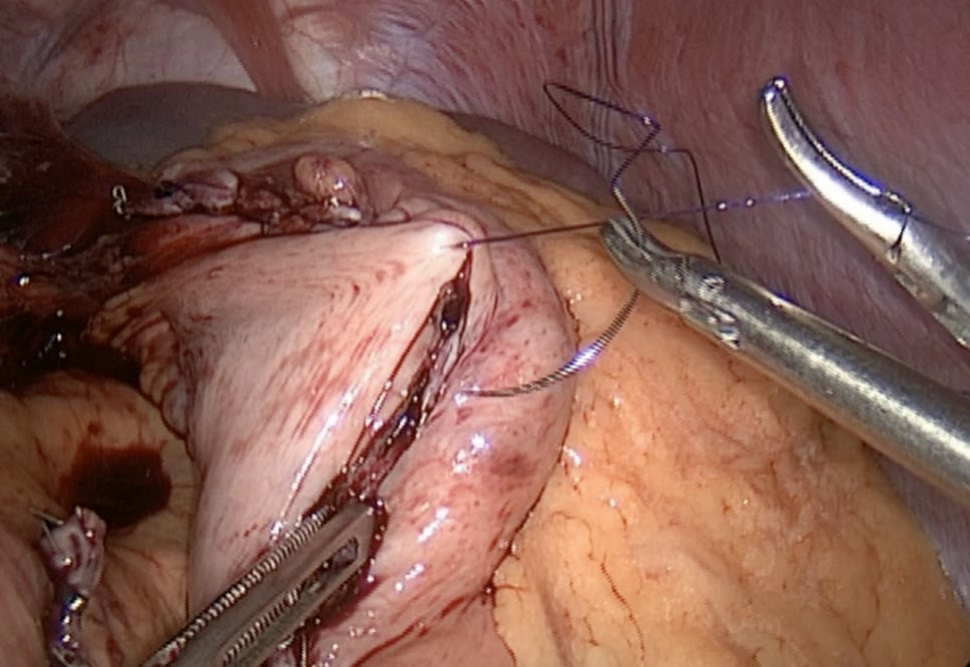
Oversewing of the gastric remnant staple line in LRYGB

The protocol designed to reduce bleeding is analyzed in three parts outlined below:

- 1. Preoperative management
  Routine preoperative preparation consists of a biochemical panel including renal and hepatic function, blood glucose and hemoglobin A1C levels, a complete blood count (CBC), coagulation profile, thyroid function tests, dexamethasone suppression test, chest X-ray, electrocardiogram, esophagogastroduodenoscopy, and abdominal ultrasonography.
  Thromboembolic prophylaxis included the administration of Enoxaparin at a dose of 6000 IU given 12 h before surgery and continued once daily. The Autologous Blood Recovery System which delivers back a patient’s own blood, known as the Cell Saver device, should be readily accessible in the operating room. A high-dependency unit bed must be available on the day of surgery, and the intensive care unit should be informed of any potential negative outcomes.
- 2. Surgical technique and intraoperative management
  All surgical procedures were performed by a single surgeon using a standardized four-trocar approach. For LSG, the procedure began with the dissection of small branches of the gastroepiploic arch, starting 6 cm from the pylorus. Dissection continued along the great curvature of the stomach, close to the gastric wall, and extended up to the short gastric vessels, which were also dissected. During omentum dissection, a double-line sealing technique with energy devices was utilized to minimize the risk of bleeding (Fig. 1), given that approximately half of the postoperative bleeding cases in LSG originate from the omentum or are of unidentified source [3]. Although vessels within the gastrocolic and gastrosplenic ligaments, which have a rich blood supply, are sealed during surgery, increased postoperative blood pressure can still induce bleeding. Moreover, in obese individuals, excessive omental fat tissue may compromise the effective application of the sealing device across vessels. In addition, the administration of antithrombotic drugs can result in bleeding from small vessels in the omentum, despite effective sealing. To mitigate the risk of omental bleeding, the use of combinations of sealing devices and hemostatic titanium clips may be considered as an option. The resection involved the use of 4–5 linear staplers (Medtronic, GIA™ Stapler with Tri-Staple™ Technology, 60 mm Medium/Thick Cartridge), guided by a 36 Fr orogastric tube. Following resection, hemostatic clips were applied for potential bleeding sites. Staple line reinforcement with omentopexy was performed using 3/0 monofilament synthetic absorbable suture (Fig. 2).
  For LRYGB, the creation of the gastric pouch involved the use of linear staples (Medtronic, GIA™ Stapler with Tri-Staple™ Technology, Medium/Thick Cartridge). The biliopancreatic and alimentary limbs were constructed to be 80 cm and 150 cm in length, respectively. An antecolic gastrojejunostomy was constructed using the same linear staples. The anastomotic opening was closed with a 3/0 V-Loc barbed suture (Medtronic, Dublin, Ireland). The enteroenterostomy was created using linear staples (Medtronic, GIA™ Stapler with Tri-Staple™ Technology, Vascular/Medium Cartridge). Hemostatic clips were applied for potential bleeding sites along the staple lines (Fig. 3). The staple lines were reinforced with 3/0 monofilament absorbable suture, including the remnant part (Fig. 4). Although applying fibrin sealant to the staple line has proven effective in reducing postoperative bleeding, it is crucial to obtain patient consent, as this treatment involves a blood product that may be rejected by the patient [7].
  Selecting the correct staple size is crucial to ensure optimal staple formation and appropriate tissue compression while avoiding risks like leakage and bleeding [8]. A brief interval of 30 s after each stapler firing was given to minimize bleeding risk [9]. We routinely maintain the patient’s blood pressure within a normal range throughout surgery and prevent hypertension by adjusting the infusion of remifentanil in all procedures. After completing the stapling and reinforcement stages, a cautious approach is taken to address potential postoperative bleeding related to blood pressure fluctuations. This involves a controlled elevation of blood pressure (140–150 systolic), a brief 5–10 min intermission in pneumoperitoneum, followed by re-insufflation and meticulous bleeding control.
  Jackson Pratt drains were routinely used in all surgeries. Fascial defects caused by 10 mm and wider trocars were closed with no 1 absorbable polyfilament suture.
- 3. Postoperative management
  The drain should be carefully monitored for hemorrhagic content and 1 g tranexamic acid should be administered in suspected bleeding. Thromboembolic prophylaxis was continued for 15 days post-surgery. In addition, venous compression stockings were applied to the lower limbs. Stress ulcer prophylaxis included intravenous administration of pantoprazole 40 mg during hospitalization followed by daily oral administration of the same dose after discharge. Complete blood count was routinely checked at postoperative 8th hour, 24th hour, and 48th hour. The liquid diet was initiated on postoperative day 1, followed by the introduction of a pureed diet on postoperative day 14, and a solid diet was initiated on postoperative day 30. Patients were discharged based on various factors, including their ability to adapt to a liquid diet, management of pain, resolution of nausea and vomiting, and overall well-being. Subsequent outpatient follow-ups were conducted on the 7th, 14th, and 30th days post-surgery. These visits included a comprehensive physical examination, including an inspection of the surgical wound sites.

## Results

From January 2019 to October 2023, 11 patients who underwent bariatric surgery at our institution explicitly refused blood and blood product transfusions. All were administered the specific protocol designed to minimize bleeding risk. Notably, each of these patients was identified as Jehovah’s Witnesses. Following the recommendations of the multidisciplinary council and the final decision of the patients, ten individuals proceeded with LSG, while one patient opted for LRYGB. The characteristics of the patients are shown in Table 1. None of the patients used anticoagulant or antiplatelet drugs preoperatively. All procedures were completed laparoscopically without any intraoperative complications.

**Table 1:** Patient characteristics

Variables	Value
Age (years)	
mean ± SD	44.4 ± 8.8
Gender	
Female, no (%)	9 (82)
Male, no (%)	2 (18)
Weight (kg), mean ± SD	112.4 ± 23.9
Preoperative BMI (kg/m^2^), mean ± SD	41.0 ± 5.1
ASA score, *n*	
2	6
3	5
Comorbidities, *n*	
Type 2 diabetes	3
Hypertension	4
Dyslipidemia	2
Obstructive sleep apnea	2
Asthma	1
Depression/anxiety disorders	2
Non-alcoholic fatty liver disease	2
Insulin resistance	2
Hepatitis B	1

No major postoperative complications, including bleeding and thrombosis, were observed. There was no need for blood product transfusion or re-intervention. All patients maintained stable and normal hemodynamics during the postoperative period. Only one patient encountered a minor complication, classified as Grade 1 by the Clavien–Dindo Classification, comprising nausea and vomiting that responded to medication. In addition, one LSG patient had 50 cc of hemorrhagic drainage in the early postoperative period. Following a 1 g infusion of tranexamic acid, the nature of the drainage transitioned to serous. Notably, this patient was not classified as bleeding, given the absence of clinical signs and the lack of a significant decrease in hemoglobin levels. The operative outcomes are shown in Tables 2 and 3.

**Table 2:** Operative outcomes of bariatric surgeries

	LSG (*n* = 10)	LRYGB (*n* = 1)
Operative time, mean (minutes)	57.1 ± 8.7	110
Estimated blood loss, mean (mL)	20	40
Length of hospital stay, mean (days)	2.5 ± 0.5	3
Tranexamic acid usage, *n*	1	0
Drainage volume (first 24 h), mean (mL)	72.5 ± 21.9	80
30-day complications, *n*		
Nausea and vomiting	1	0

**Table 3:** Patient follow-up with blood tests

	LSG 1	LSG 2	LSG 3	LSG 4	LSG 5	LSG 6	LSG 7	LSG 8	LSG 9	LSG 10	LRYGB
Hemoglobin (g/dL)											
Preoperative	12	12	12	14	12	13	12	12	12	11	11.5
Day 0	6	7	11	12	1	2	4	3	1	10	11.2
Day 1	12.1	12.2	11.4	11.7	12.2	12.7	11.8	11.4	11.3	10.1	11.4
Day 2	11.8	11.8	11.1	11.9	12	13.3	12	10.9	11.8	9.2	11.3
Hematocrit (%)											
Preoperative	37.2	36.9	37	40.5	40.3	38.5	40	36.9	36.6	34	35.5
Day 0	37	36	36	38	37.4	37	39.2	34.5	36	31.6	34.8
Day 1	36.8	34.9	35.8	37.4	37.5	36.5	39	34.4	35.4	32	35.2
Day 2	36.4	34.8	35	37.7	37.4	39.6	39.1	33	35.7	30	35
Drainage (mL)											
Day 1	50	50	75	100	50	75	75	100	50	100	80
Day 2	25	25	25	60	50	50	25	60	30	50	40
Tranexamic acid usage	–	–	–	+	–	–	–	–	–	–	–

A patient scheduled for LSG presented with severe iron deficiency anemia, indicated by a hemoglobin level of 6.9 g/dL. The patient underwent intravenous iron treatment, and surgery proceeded once the hemoglobin level increased to 11 g/dL.

## Discussion

This report presents the outcomes of 11 JW patients undergoing bariatric surgery, featuring a unique aspect by including 10 consecutive LSG patients. In this series of ASA class 2 and 3 patients, no major complications such as bleeding, hematoma, thromboembolism, or leakage were observed in any patient.

Only one original article and one letter to the editor were identified in the MEDLINE database when searching for the keywords “Jehovah” or “bloodless surgery” in combination with terms related to bariatric surgery. Kitahama et al. presented a series of 35 patients undergoing LRYGB and Laparoscopic Adjustable Gastric Band (LAGB) [5]. In their series, all operations were completed laparoscopically, and only one LRYGB patient experienced hemorrhage due to liver laceration, requiring reoperation. The patient, with a postoperative hematocrit drop to 19.6, was successfully managed with erythropoietin and iron injections, ultimately being discharged on postoperative day 5. No instances of thromboembolism or death were reported. It is noteworthy that LSG, the most common bariatric surgery performed today, was not encountered in Kitahama’s study. In a letter published in December 2023, a series involving nine patients was described, including four who underwent LSG and three who underwent LRYGB. All patients were discharged uneventfully at the postoperative 48th hour without bleeding. Also, the letter presented a protocol designed to minimize bleeding in bariatric surgery JW patients [6]. These studies suggest that bariatric surgeries can be safely performed in groups of patients who refuse blood transfusions, without any concerns.

The patients refusing blood products have been addressed in various surgical contexts, including cardiac, major abdominal, gynecologic, orthopedic, maxillofacial surgeries, and organ transplantation. These studies indicate that JW and non-JW patients have similar perioperative outcomes with appropriate management strategies [10].

## Conclusion

Bariatric surgery can be performed with acceptable complication rates in patients refusing blood transfusions, such as Jehovah's Witnesses, by implementing established protocols aimed at enhancing patient safety.

## Conflict of interest

The authors declare that they have no conflict of interest.
